# Inhibition of integrin α_V_β_6_ changes fibril thickness of stromal collagen in experimental carcinomas

**DOI:** 10.1186/s12964-018-0249-7

**Published:** 2018-07-02

**Authors:** P. Olof Olsson, Renata Gustafsson, Alexei V. Salnikov, Maria Göthe, Kathrin S. Zeller, Tomas Friman, Bo Baldetorp, Louise A. Koopman, Paul H. Weinreb, Shelia M. Violette, Sebastian Kalamajski, Nils-Erik Heldin, Kristofer Rubin

**Affiliations:** 1 Department of Experimental Medical Science, Medicon Village 406, SE-22381 Lund, Sweden; 2 Oncology Clinic, Department of Clinical Sciences, University Hospital Lund, SE-221 85, Lund, Sweden; 3 Science for Life Laboratories, Department of Medical Biochemistry and Microbiology, Uppsala University, BMC, Box 582, SE-751 23 Uppsala, Sweden; 4 Biogen, 225 Binney Street, Cambridge, MA 02142 USA; 5 Department of Immunology, Genetics and Pathology, The Rudbeck Laboratory, Uppsala University, Uppsala, Sweden

## Abstract

**Background:**

Chemotherapeutic efficacy can be improved by targeting the structure and function of the extracellular matrix (ECM) in the carcinomal stroma. This can be accomplished by e.g. inhibiting TGF-β1 and -β3 or treating with Imatinib, which results in scarcer collagen fibril structure in xenografted human KAT-4/HT29 (KAT-4) colon adenocarcinoma.

**Methods:**

The potential role of α_V_β_6_ integrin-mediated activation of latent TGF-β was studied in cultured KAT-4 and Capan-2 human ductal pancreatic carcinoma cells as well as in xenograft carcinoma generated by these cells. The monoclonal α_V_β_6_ integrin-specific monoclonal antibody 3G9 was used to inhibit the α_V_β_6_ integrin activity.

**Results:**

Both KAT-4 and Capan-2 cells expressed the α_V_β_6_ integrin but only KAT-4 cells could utilize this integrin to activate latent TGF-β in vitro. Only when Capan-2 cells were co-cultured with human F99 fibroblasts was the integrin activation mechanism triggered, suggesting a more complex, fibroblast-dependent, activation pathway. In nude mice, a 10-day treatment with 3G9 reduced collagen fibril thickness and interstitial fluid pressure in KAT-4 but not in the more desmoplastic Capan-2 tumors that, to achieve a similar effect, required a prolonged 3G9 treatment. In contrast, a 10-day direct inhibition of TGF-β1 and -β3 reduced collagen fibril thickness in both tumor models.

**Conclusion:**

Our data demonstrate that the α_V_β_6_-directed activation of latent TGF-β plays a pivotal role in modulating the stromal collagen network in carcinoma, but that the sensitivity to α_V_β_6_ inhibition depends on the simultaneous presence of alternative paths for latent TGF-β activation and the extent of desmoplasia.

**Electronic supplementary material:**

The online version of this article (10.1186/s12964-018-0249-7) contains supplementary material, which is available to authorized users.

## Background

Carcinomal stroma shares many characteristics with a healing wound or with a chronic inflammatory lesion. Commonly observed stromal features are leukocyte infiltration, fibrin deposition, increased blood vessel permeability for plasma proteins, and increased production and metabolism of ECM proteins [1, 2]. Malignant and normal stromal cells, including infiltrating inflammatory cells, participate in reciprocal signaling that results in the buildup of the carcinomal stroma [2, 3]. Also, both human and experimental carcinomas have a pathologically elevated intratumoral interstitial fluid pressure (P_IF_) [4]. Several P_IF_-lowering agents increase the uptake and efficacy of small molecular weight chemotherapeutic agents in experimental carcinomas [4] suggesting that an elevated P_IF_ reflects impediment of solute transport into carcinoma tissue.

Carcinoma-associated fibroblasts (CAFs) originate from phenotypic switches of infiltrated or resident mesenchymal cells and may also originate from epithelial-to-mesenchymal transition of carcinoma cells. This process can be triggered by soluble mediators secreted by malignant cells or by infiltrating leukocytes [3, 5]. CAFs or a CAF subpopulation is the major producer of the stromal collagen fiber network and the other ECM components. In many aspects CAFs resemble “activated” fibroblasts in healing wounds and in fibrotic lesions. The formation of CAFs is modulated by TGF-β [6] that also stimulates collagen production and constitutes a key factor in the development of fibrosis [7, 8]. The three TGF-β isoforms, TGF-β1, −β2 and -β3, are secreted as small latent complexes (SLC) in which the active growth factors are non-covalently bound to latency associated peptides (LAPs) (for a recent review see: [9]). During maturation in the endoplasmic reticulum most of the nascent SLC becomes covalently bound via disulfide bonds to latent TGF-β binding proteins (LTBP), forming the large latent complex (LLC) that covalently associates with the ECM [9]. Since latent TGF-β complexes are inactive, a TGF-β release mechanism is necessary in order for the cytokine to bind and stimulate its cognate receptors. The known release mechanisms include the action of reactive oxygen species, extreme acidification, proteolytic cleavage, interaction with thrombospondin, or conformational change of the latent complex, [9]. Among the conformational change release mechanisms the integrins α_V_β_6_ and α_V_β_8_ have been extensively described [9]. Data from studies with genetically modified mice suggest that integrin-directed activation of latent TGF-β complexes plays a major role in TGF-β dependent processes in vivo [10, 11]. The integrin α_V_β_6_ is implicated in development [10] and in cancer [12, 13], its expression is restricted to cells of epithelial origin, and it is upregulated in many cancer types and can be used as a prognostic factor [14]. α_V_β_6_ promotes carcinoma growth and invasion [13] and can act pro-migratory in pancreatic cancer cells [15] in addition to its TGF-β activating function that is important also for matrix metalloproteinase expression [16].

Previously, we have shown that human KAT-4 carcinoma cells transfer TGF-β to fibroblasts via intercellular contacts, resulting in an increased collagen type I protein synthesis [17]. In vivo, treatment with the soluble TGF-β type II receptor-murine Fc:IgG_2A_ fusion protein (Fc:TβRII), a specific inhibitor of TGF-β1 and -β3, reduces intratumoral collagen density and influences the collagen network architecture in xenograft human KAT-4 cell-derived carcinoma [18]. In addition, Fc:TβRII lowers intratumoral P_IF_, dampens the expression of inflammation-related genes, reduces vascular protein leakage, and increases the treatment efficacy of doxorubicin [19]. Here we investigated the importance of α_V_β_6_ integrin for the collagen network ultrastructure in the previously characterized model of colorectal carcinoma, KAT-4, and in a model of desmoplastic pancreatic cancer, Capan-2 [20].

## Methods

### Reagents

The function-blocking anti-α_V_β_6_ antibody 6.3G9 (henceforth referred to as 3G9) has been described previously [21]. The TGF-β receptor type II-murine Fc:IgG_2A_ chimeric protein (Fc:TβRII) that functionally inhibits TGF-β1 and -β3, as well as control IgG_1_ clone 1E6, were all prepared sterile and pyrogen-free at Biogen and used as solutions of the purified proteins in PBS and stored at − 80 °C until use. The c-RGD peptides cyclo-(Arg-Gly-Asp-D-Phe-Val), a selective inhibitor of the α_V_β_3_ integrin [22], was from Bachem (Bubendorf, Switzerland) (H-2574) and Cilengitide, a selective inhibitor of the α_V_β_3_ and α_V_β_5_ integrins [23] was from Selleckchem (Munich, Germany). The ROCK inhibitor Y-27632 was from Sigma-Aldrich. Human fibronectin and vitronectin were purified from fresh human plasma as described [24, 25].

### TGF-β assay

The quantitative PAI-1/luciferase bioassay for TGF-β [21, 26] was used to investigate the production of TGF-β by carcinoma cells in vitro. TGF-β induces the expression of plasminogen-activator inhibitor-1 (PAI-1) in Mink Lung Epithelial cells (MLEC, clone C32) transfected with a truncated PAI-1 promoter fused to the firefly luciferase reporter gene. For co-culture assays 96- or 24-well plates for transwell assays (Corning® HTS Transwell®; Sigma Aldrich Sweden AB, Stockholm, Sweden) were pre-coated 3 h or overnight with DMEM containing 10% FBS. MLEC were seeded in DMEM supplemented with 0.1% BSA (Sigma Aldrich) and allowed to attach for 3 h. KAT-4 and Capan-2 cells were then added to the wells or into the transwell inserts. KAT-4 and Capan-2 carcinoma cell s were incubated with 3G9, Fc:TβRII or control IgG_1_ for 30 min on ice before they were added to the MLEC. Plates were incubated at 37 °C for 20 h. Cells and the conditioned medium were harvested and analyzed according to the manufacturer’s protocol (Luciferase Assay System; Promega Biotech AB, Nacka, Sweden). Capan-2 cells were pre-acclimatized to growth in DMEM when to be co-cultured with fibroblasts. F99 normal human fibroblasts were isolated from explant cultures of human skin from patients who underwent breast reduction surgery. MLEC (0.25 × 10^4^ cells) and a combined 0.25 × 10^4^ of malignant cells or fibroblasts were seeded per well in 96 well plates.

### Animals

Athymic C57BL nu/nu female mice were purchased from Taconic Europe (Ry, Denmark) and were between 6 and 10 weeks of age. Prior to the inoculation of carcinoma cells mice were acclimatized at the local animal facility. Animals were individually identified by ear markings. All animal experiments were performed at the animal facilities of the National Veterinary Institute and Lund University, Sweden, in accordance with approval by the ethical committees for animal experiments in Uppsala and Malmö/Lund, Sweden. The number of animals was minimized to comply with guidelines from the Ethical Committee and EU legislation on the use of laboratory animals.

### Cells/xenograft models

KAT-4 cells were originally described as originating from a thyroid tumor [27], but a thyroid origin of the KAT-4 carcinoma has been questioned and the cells have been identified as part of the human colorectal adenocarcinoma cell line HT-29 [28]. Human pancreatic carcinoma 139/Capan-2/JE cells were from ATCC. The identities of the two cell lines were ascertained by Short Tandem Repeat loci analyses (IdentiCell, Aarhus, Denmark). Capan-2 showed a 100% match, whereas KAT-4, as expected [28], matched with HT-29 although alleles D13S317:12 and TH01:9 were absent. Normal human dermal F99 fibroblasts that had been isolated as described [29] were kindly donated by prof. Bengt Gerdin (Uppsala University). KAT-4 cells and F99 fibroblasts were grown in Dulbecco’s modified Eagle’s medium (DMEM; Gibco Life Technologies, Stockholm, Sweden) and Capan-2 cells were grown in McCoy’s 5A medium with GlutaMax (Gibco), both supplemented with 10% FBS (Gibco) and antibiotics. In the protocol used in Uppsala 50 μL of cell suspensions containing either 2 × 10^6^ KAT-4 cells or 5 × 10^6^ Capan-2 cells were injected *s.c.* in the left flank of the mice. Mice that developed carcinomas were inspected every other day and the external sizes (length x width x height) of the carcinomas were measured using a vernier caliper.

### Treatment with 3G9 and fc:TβRII

Treatments were initiated when KAT-4 and Capan-2 tumors reached a size of approximately 100 mm^3^. Tumor treatment groups were size-matched and animals were treated with PBS or control IgG_1_, 3G9 IgG, or Fc:TβRII via intra-peritoneal injection in a maximal volume of 100 μL. 3G9 IgG was administered at doses of 3, 10 and 30 mg/kg and Fc:TβRII at a dose of 10 mg/kg at days 1 and 7 in experiments with KAT-4 carcinomas. Capan-2 carcinomas were treated with 10 mg/kg of 3G9 IgG or Fc:TβRII at days 1, 3 and 7. Animals were sacrificed and carcinomas collected at day 11. For some experiments Capan-2 carcinomas were treated according to an extended protocol with 10 mg/kg 3G9 IgG administered at days 1, 3, 7, 10 and 14 and carcinomas collected at day 19 (extended protocol).

### Measurement of interstitial fluid pressure

Tumor P_IF_ was measured by the “wick-in needle” technique as we have previously described [30]. For data recording and collection the Microsoft Excel, ADI PowerLab (Software Chart v. 4.1 and Scope v. 3.6.8 for Macintosh) was used.

### Immunohistochemistry and histology

Tumors were dissected, embedded in OCT (Sakura) and frozen at − 80 °C. Cryosections (6–8 μm) were fixed in acetone at 8 °C, blocked in 20% goat serum (Serotec, Oxford, UK), incubated with monoclonal rat anti-mouse PECAM-1 (CD31) clones Mec 13.3 and 390 (BD, San Diego, CA, USA) and incubated with goat anti-rat biotinylated IgG (Vector laboratories, Burlingame, CA, USA). To visualize α_V_β_6_ in tissue sections purified 3G9 was biotinylated with N-hydroxysuccinimide biotin ester overnight at 4 °C, followed by a single desalting step with PD-10 columns in PBS with 0.1% BSA and 0.02% azide as preservative. Sections were blocked in 5% swine serum (Sigma) and 2% BSA (Sigma), incubated with biotinylated 3G9 antibody and then with Fluorescein Avidin D (Vector Labs). Slides were mounted with Vectamount containing DAPI (Vector Labs), and the staining was analyzed using a Nikon Eclipse 80i microscope (Nikon Instruments, Japan).

For histological assessment, Masson’s trichrome and Sirius red staining was performed using the Accustain Masson Trichrome stain (Sigma-Aldrich) and 0.1% Picrosirius Red solution (Solveco, Sweden) according to the manufacturer’s instructions. Hematoxylin and eosin staining was performed according to standard protocols.

### Determination of vessel density

CD31 immunostaining was performed on KAT4 and Capan-2 carcinomas treated with 3G9 or PBS. Sections were then analyzed after imaging. CD31- positive structures, having an area greater or equal to that of the smallest discernible vessel (29 μm^2^), were counted and plotted per mm^2^. All comparisons were done from sections stained simultaneously, with the same exposure for image capture, and a defined threshold for image analysis. Images were analyzed in ImageJ software (NIH, Bethesda, Maryland, USA).

### Hydroxyproline measurement

KAT4 carcinomas were hydrolyzed in 6 M HCl for 4 h at 120 °C at a pressure of 2 atm. Hydroxyproline content in the hydrolysates was determined essentially as previously described [31].

### Electron microscopy

For transmission electron microscopy analyses were fixed in 0.15 M sodium cacodylate-buffered 2% glutaraldehyde, post-fixed in 0.15 M sodium cacodylate-buffered 1% osmium tetraoxide, dehydrated in graded ethanol series, impregnated in acetone and embedded in epoxy resin. Ultrathin sections were examined in a Philips CM-10 electron microscope (Philips, Amsterdam, Netherlands) [32]. Collagen fibril diameter was measured using ImageJ software (NIH, Bethesda, Maryland, USA).

### Flow cytometry

Cells (5 × 10^6^) were incubated with the anti-α_V_β_6_ antibody 3G9 or mouse IgG as a negative control in PBS for 1 h, washed and incubated with FITC-conjugated rabbit anti-mouse IgG (DAKO, Glostrup, Denmark) secondary antibody for 45 min, washed and suspended in FACS buffer. A titration was done with both primary and secondary antibodies to determine the appropriate amount, which was 0.12 mg/mL 3G9 and 1:100 dilution of the secondary antibody. The flow cytometry analysis was performed using a FACSAria (Becton Dickinson).

### RNA extraction and Illumina gene expression analysis

Total RNA from 3-dimensional free floating collagen gels was extracted with the Direct-zol RNA MiniPrep Kit (Zymo Research, Irvine, CA) according to the manufacturers’ instructions. RNA was quantified with Nanodrop (Thermoscientific), integrity-validated on Bioanalyzer, and run on an Illumina platform (SCIBLU, LTH, Lunds University). Signal intensities were first filtered using internal standards, then by normalization to the observed background level. Collagen gels were made in 48-well plates, coated for 2 h with 2% BSA (Sigma) at 37 °C. Carcinoma cells were re-suspended to a concentration of 1 × 10^6^ cells/ml and mixed 1:9 with a freshly prepared collagen gel (50% 2xDMEM, 10% 200 mM HEPES pH 8 and 40% 3.1 mg/ml type I bovine collagen (Biomatrix)). Gels were allowed to solidify at 37 °C for 2 h before being floated with serum free DMEM media and incubated at 37 °C 5% CO_2_ for 48 h. Gels were dissolved and stored in Trizol (Thermofisher) for later total RNA extraction.

### Microarray data analysis

Illumina Microarray (SCIBLU) data was initially pre-processed and normalized using the Quantile Normalization method [33]. Analyses were performed using GenomeStudio software V2011.1. The data have been deposited in NCBI’s Gene Expression Omnibus and are accessible through GEO Series accession number GSE85255 (https://www.ncbi.nlm.nih.gov/geo/query/acc.cgi?acc=GSE85255). Non-annotated probe sets and probe sets with signal intensities below the median level of negative control intensities, in 20% of the samples, not belong to one condition, were excluded. Differential expression analysis was performed using DESeq2 script from Bioconductor [34].

### Statistical methods

Unpaired, two-tailed Student’s *t*-test and the Mann-Whitney *U* test were used and *p* < 0.05 was considered statistically significant. The standard deviations of data points are indicated in the figures. All statistics were performed in GraphPad Prism 6 (La Jolla, CA, USA). All in vitro data was collected from a minimum of three or more independent experiments.

## Results

### KAT-4 and Capan-2 human carcinoma cells produce and secrete latent TGF-β

**Fig. 1:**
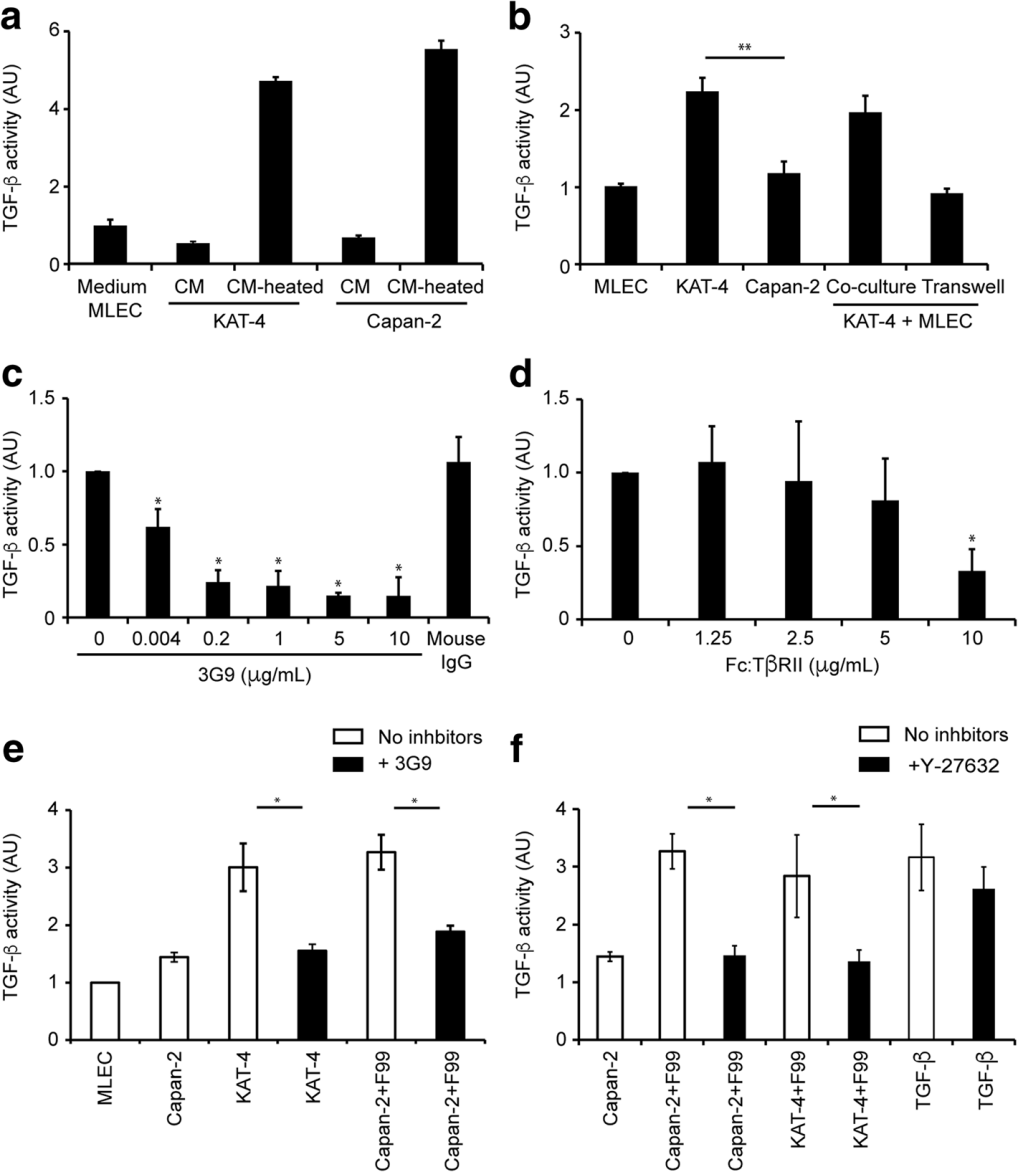
Luciferase-based assay showing TGF-β activity in mink lung epithelial cells (MLEC). **a** Effects by TGF-β elicited signaling in MLEC by heat-activated (CM heated) or non-activated (CM) conditioned medium from KAT-4 and Capan-2 cell cultures. **b** TGF-β activity in cultures of MLEC, KAT-4, Capan-2, and co-cultures of KAT-4 and MLEC (*p* = 0.0014). **c** Effect of 3G9 antibody on TGF-β activity in co-cultures of MLEC and KAT-4 cells**. d** Effect of Fc:TβRII on TGF-β activity in co-cultures of MLEC with KAT-4 cells. **e** Effect of 3G9 on TGF-β activity in KAT-4 and MLEC co-cultured with F99 fibroblasts and Capan-2 cells. **f** Effects of the ROCK inhibitor Y-27632 (10 μM) on TGF-β activity in fibroblasts co-cultured with tumor cells, as well as effects of TGF-β supplementation (10 nM). All data points were compiled from a minimum of three independent experiments performed in triplicates. Error bars are standard deviations. Asterisks indicate *p* < 0.05 as analyzed with Student’s t-test

Conditioned media (CM) from cultures of KAT-4 and Capan-2 cells were collected and analyzed to measure total TGF-β levels using luciferase-based MLEC assay. Cell culture medium from MLEC was used as a negative control. Heat-activated CM from KAT-4 and Capan-2 cell cultures elicited PAI-1 activity (TGF-β signaling) in the reporter cells, whereas the corresponding non-activated CM had no effect (Fig. 1a). Heat-activated CM from Capan-2 and KAT-4 cell cultures contained similar amounts of TGF-β. The results show that both KAT-4 and Capan-2 carcinoma cells produced and secreted latent TGF-β.

Next, we elucidated whether KAT-4 and Capan-2 cells were able to activate latent TGF-β when co-cultured with MLEC allowing for the formation of direct cell-cell contacts. Active TGF-β was transferred from KAT-4 to the adjacent MLEC when the two cell types were co-cultured (Fig. 1b). When KAT-4 cells were separated from the MLEC cells by a porous membrane that allowed free passage of growth factors (Transwell system) no TGF-β signaling was detected in the MLEC. Capan-2 cells lacked the ability to activate the growth factor also when co-cultured with the reporter MLEC (Fig. 1b).

### Cultured carcinoma cells activate latent TGF-β by an integrin α_V_β_6_-dependent mechanism

To validate that KAT-4 and Capan-2 cells express similar amounts of cell surface integrin α_V_β_6_ we used flow cytometry and the anti-integrin α_V_β_6_ specific monoclonal antibody 3G9 (Additional file 1). 3G9 IgG effectively inhibited TGF-β elicited signaling in MLEC co-cultured with KAT-4 cells over a wide range of IgG concentrations (0.004 to 10 μg/mL) demonstrating the involvement of integrin α_V_β_6_ in KAT-4 cell-driven activation of latent TGF-β (Fig. 1c). Furthermore, the specific TGF-β1 and -β3 inhibitor Fc:TβRII (10 μg/mL) inhibited TGF-β elicited signaling in MLEC co-cultured with KAT-4 cells, demonstrating that KAT-4 cells produce and elicit a TGF-β signaling in the target MLEC cells through TGF-β1 and/or -β3 (Fig. 1d). Addition of 3G9 or Fc:TβRII to co-cultures of MLEC and Capan-2 cells had no effect on TGF-β signaling (Fig. 1e). Normal human dermal F99 fibroblasts co-cultured with MLEC were unable to activate latent TGF-β but active TGF-β was generated when F99 fibroblasts, Capan-2 cells and MLEC were co-cultured; this activation was inhibited by 3G9 (Fig. 1e). To further control for integrin specificity we investigated the effects of cilengitide, an Arg-Gly-Asp containing peptide that inhibits the α_V_β_3_ and α_V_β_5_ integrins in nM ranges, and the α_V_β_3_ selective antagonist peptide cRGD. Neither of the peptides inhibited the activation of latent TGF-β by Capan-2/F99 cells over a wide concentration range, suggesting that α_V_β_3_ and α_V_β_5_ integrins were dispensable for the activation process (Additional file 2). Finally, to investigate whether Capan-2 cells alone are unable to activate latent TGF-β due to insufficient amounts of auxiliary proteins, such as fibronectin [35], we cultured Capan-2 cells on dishes pre-coated with human plasma fibronectin or vitronectin. Neither condition rendered Capan-2 cells capable of activating latent TGF-β (Additional file 2).

Rho kinases and the down-stream effector ROCK play a pivotal role in α_V_β_6_-mediated activation of latent TGF-β by epithelial cells [36]. We therefore investigated the effects of the selective ROCK I and II inhibitor Y-27632 on carcinoma cell-driven activation of latent TGF-β. Supplementing the cell cultures with 10 μM Y-27632 effectively inhibited the activation of latent TGF-β by both KAT-4 and Capan-2 cells co-cultured with F99 fibroblasts (Fig. 1f). Y-27632 had, however, no effect on signaling elicited by active TGF-β resulting in increased transcription of PAI-1 in MLEC (Fig. 1f).

### Gene expression profiles of KAT-4 and Capan-2 cells cultured in three-dimensional collagen gels

**Fig. 2:**
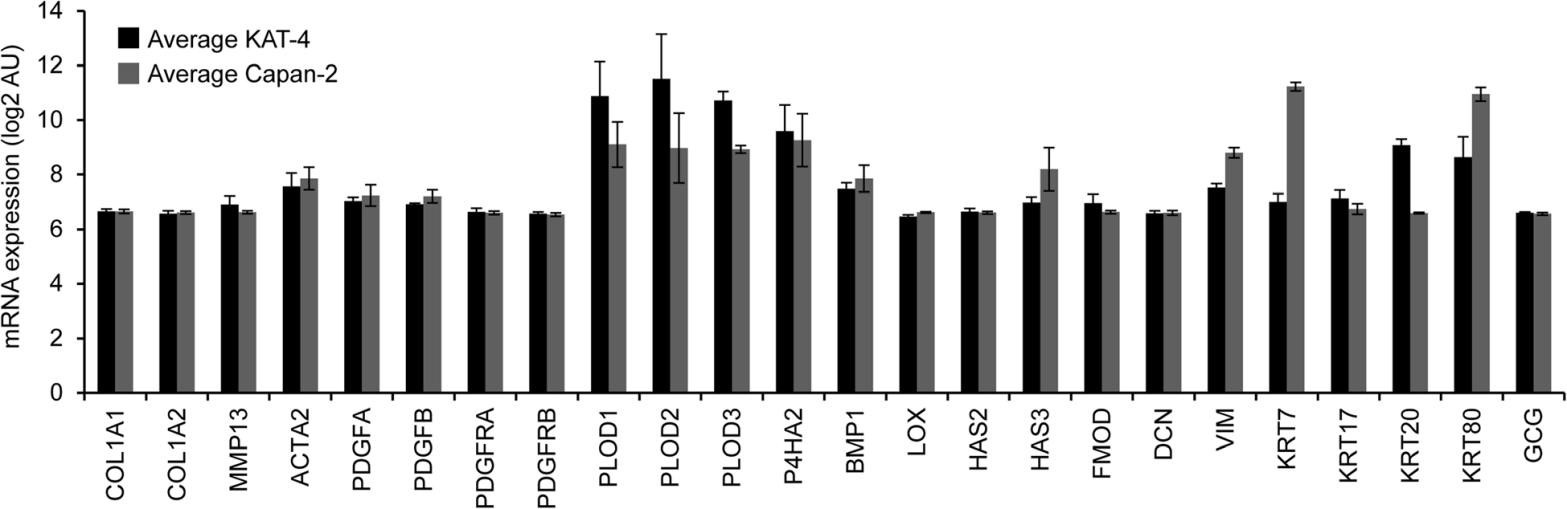
Comparison of gene expression in untreated KAT-4 (*n* = 3) and Capan-2 (*n* = 3) cells grown in three-dimensional collagen gels. mRNA expression (2log AU) of selected genes for ECM related transcripts in 3-dimensional collagen gels containing KAT-4 (black bars) and Capan2 (grey bars) Collagen α1(I) (Col1A1), collagen α2(I) (Col1A2), Matrix metalloprotease-13 (MMP-13), α2 smooth muscle actin (ACTA2), Platelet Derived Growth Factor A and B (PDGF-A and -B and PDGF receptor–α and -β (PDGFR –A and -B), procollagen-lysine, 2-oxoglutarate 5-dioxygenase − 1, − 2 and − 3 (PLOD -1, − 2 and − 3), Prolyl 4-hydroxylase subunit α-2 (P4HA2), Bone morphogenetic protein 1 (BMP-1), Lysyl oxidase (LOX), Hyaluronan Synthase − 2, and − 3 (HAS-2 and -3), Fibromodulin (FMOD), Decorin (DCN), Vimentin (VIM), Keratin-7,-17,-20 and − 80 (KRT-7,-17,-20 and − 80), Glucagone (GCG). Data was analyzed with Student’s t-test. Error bars are standard deviations

To elucidate the differences that influence the dependence on the α_V_β_6_-mediated latent TGF-β activation we performed comparisons of gene expression profiles in KAT-4 and Capan-2 cells grown in free-floating collagen gels. Illumina microarray platform analysis revealed that neither of the cell lines expressed transcripts characteristic of epithelial-to- mesenchymal transition, including genes encoding a set of ECM, ECM-modifying enzymes, cytoskeletal proteins, and PDGFs and PDGF receptors (Fig. 2). The expression signals of the glucagon gene (GCG), which is specifically expressed by α-cells of the islets of Langerhans, are shown to indicate background expression levels (Fig. 2). Thirty-four transcripts were differentially expressed in KAT-4 vs. Capan-2 cells, at cut-offs of log_2_ 4-fold difference, significance level < 0.05 and Q-value (Additional file 3). Among the keratin genes KRT20 was highly expressed in KAT-4 cells, and KRT7 was expressed in Capan-2 cells, which is in line with a colonic origin of KAT-4 cells and a ductal lining origin of Capan-2 cells. Among the extracellular space-related transcripts Capan-2 cells had upregulated tissue plasminogen activator (*PLAT*) and urokinase-type plasminogen activator (*PLAU*), and also transcripts encoding ECM protein cross-linking enzymes (*TGM2* and *LOXL4*). KAT-4 cells expressed higher levels of cell adhesion-related *TSPAN8* and *BSG.* Both cell lines had upregulated number of different metabolism- and immune response-related transcripts.

### Comparison of KAT-4 and Capan-2 carcinomas

**Fig. 3:**
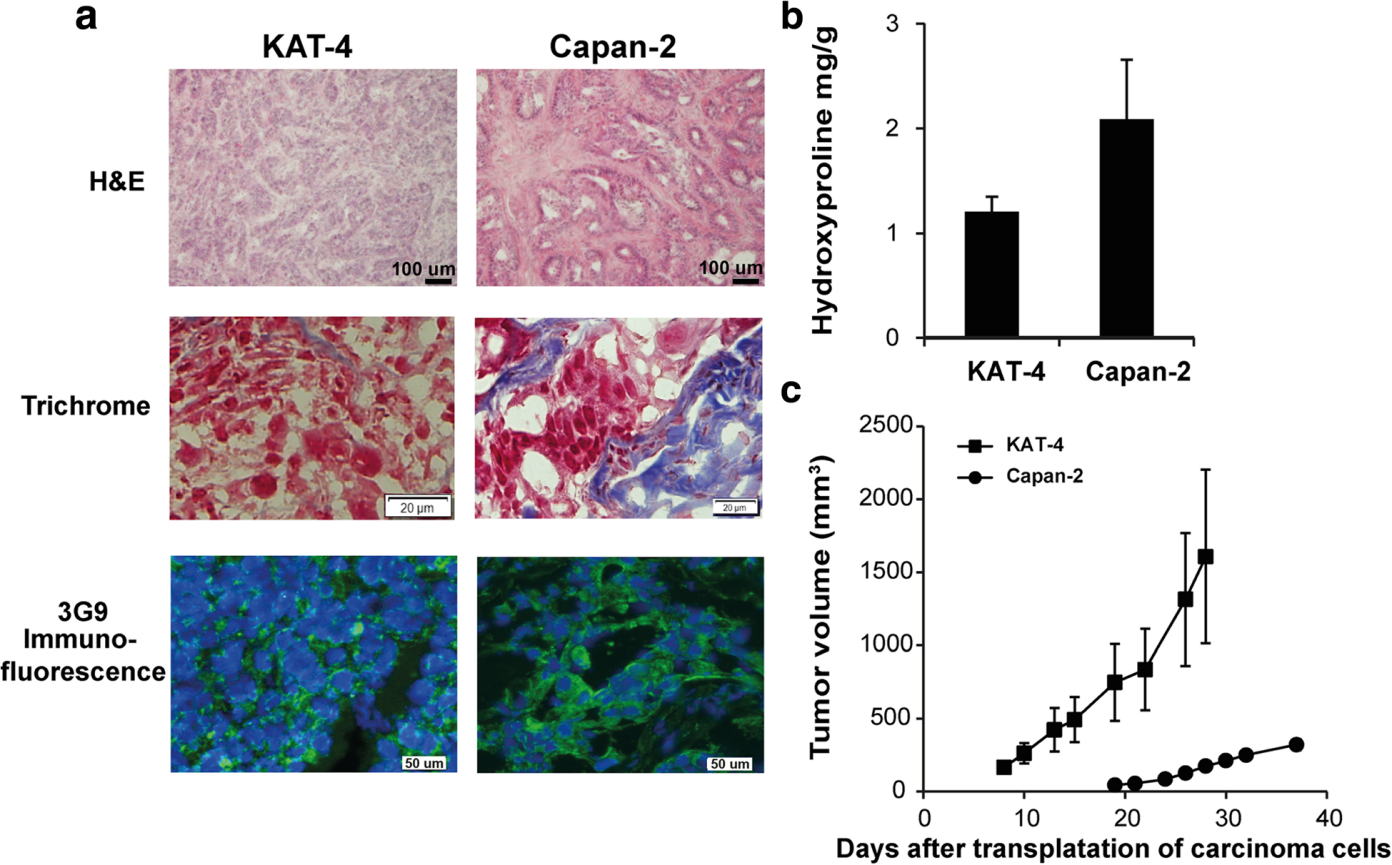
Comparison of untreated KAT-4 and Capan-2 carcinoma models. **a** Hematoxylin and Eosin and Sirius red staining in KAT-4 and Capan-2 carcinomas (bars = 100 μm). Trichrome staining (bars = 20 μm) and immunofluorescence staining with biotinylated 3G9 antibody (green) shows that the expression of integrin α_V_β_6_ is located at the cell membrane in both KAT-4 and Capan-2 carcinomas (cell nuclei stained with DAPI, blue; bars = 50 μm). **b** Collagen content in untreated KAT-4 (*n* = 4) and Capan-2 (*n* = 5) carcinomas, represented by hydroxyproline mg/g wet weight. **c** Average growth of untreated KAT-4 (*n* = 8) and Capan-2 tumors (*n* = 7), represented in mm^3^ measured externally (length x width x height)

Experimental carcinomas generated after xenografting KAT-4 or Capan-2 cells into nude mice were analyzed for morphology and collagen content. The respective carcinomas, grown to similar sizes, differed both in carcinoma cell density and in ECM structure (Fig. 3a). Capan-2 carcinomas were substantially richer in collagenous ECM and had lower cell density (Fig. 3a, b). Biotinylated 3G9 bound to epithelial cells both in KAT-4 and Capan-2 carcinomas, suggesting maintained integrin α_V_β_6_-expression during in vivo growth (Fig. 3a). Finally, in vitro-cultured KAT-4 cells grew at a faster rate than Capan-2 cells (data not shown) and this difference was reflected in tumor growth in vivo*,* i.e. KAT-4 carcinomas grew faster than Capan-2 carcinomas (Fig. 3c).

### Blockage of integrin α_V_β_6_ reduces collagen fibril diameter in KAT-4 and Capan-2 carcinomas

**Fig. 4:**
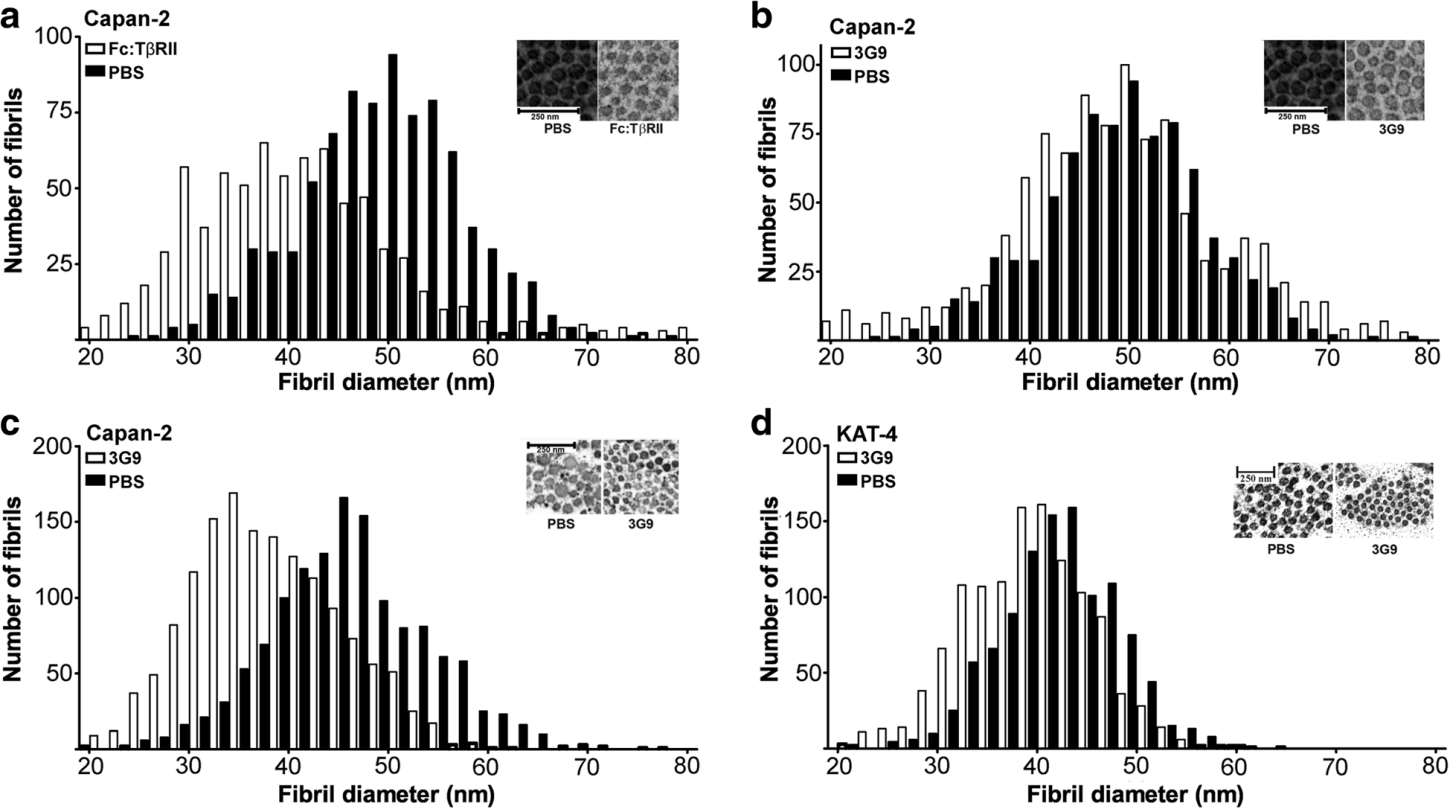
Ultrastructural analysis of KAT-4 and Capan-2 tumors using transmission electron microscopy. **a** Collagen fibril diameter in Capan-2 tumors treated with Fc:TβRII (3 × 10 mg/kg) (*n* = 5). Mean diameter was 48 nm in PBS-treated (*n* = 5) and 39 nm in 3G9-treated tumors (*p* < 0.01). **b** Collagen fibril diameter in Capan-2 tumors treated with 3G9 (3 × 10 mg/kg) (*n* = 5). No significant differences (*p* > 0.5) were observed in collagen fibril diameter after 3G9 treatment (mean diameter was approximately 49 nm in both phenotypes). **c** Collagen fibril diameter in Capan-2 tumors treated with 3G9 (5 × 10 mg/kg, extended protocol). Mean fibril diameter was 47 nm in PBS-treated and 41 nm in 3G9-treated tumors (*p* < 0.02). **d** Collagen fibril diameter in KAT-4 carcinomas treated with 3G9 (2 × 3 mg/kg) (*n* = 3). Mean fibril diameter was 43 nm in PBS-treated and 38 nm in 3G9-treated tumors (*p* = 0.02) (*n* = 3). Inserts show representative micrographs of collagen fibrils. To analyze fibril distribution, each data point (diameter for each fibril) was tabulated in GraphPad using the Frequency distribution analysis tool. Data was analyzed with Student’s t-test

Transmission electron microscopy analysis revealed that collagen fibrils in Capan-2 were on average 4 nm thicker than in KAT-4 carcinomas; they were also skewed towards thicker fibrils (compare untreated samples (black bars) in e.g. Fig. 4b, d). Treatment of Capan-2 carcinomas with Fc:TβRII at 10 mg/kg at days 1, 3 and 7 significantly reduced collagen fibril thickness (Fig. 4a). These data demonstrate a dependence of TGF-β signaling for stromal collagen network architecture in this tumor model. By contrast, fibril thickness was not affected after a 10-day treatment with 3G9 at 10 mg/kg (Fig. 4b); fibril thickness did, however, decrease significantly following an extended treatment protocol (injections at days 1, 3, 7, 10 and 14) (Fig. 4c). In contrast, a 10-day treatment of KAT-4 carcinomas with 3G9 was sufficient to significantly reduce the mean collagen fibril thickness (Fig. 4d).

### Blockage of integrin α_V_β_6_ reduces interstitial fluid pressure

**Fig. 5:**
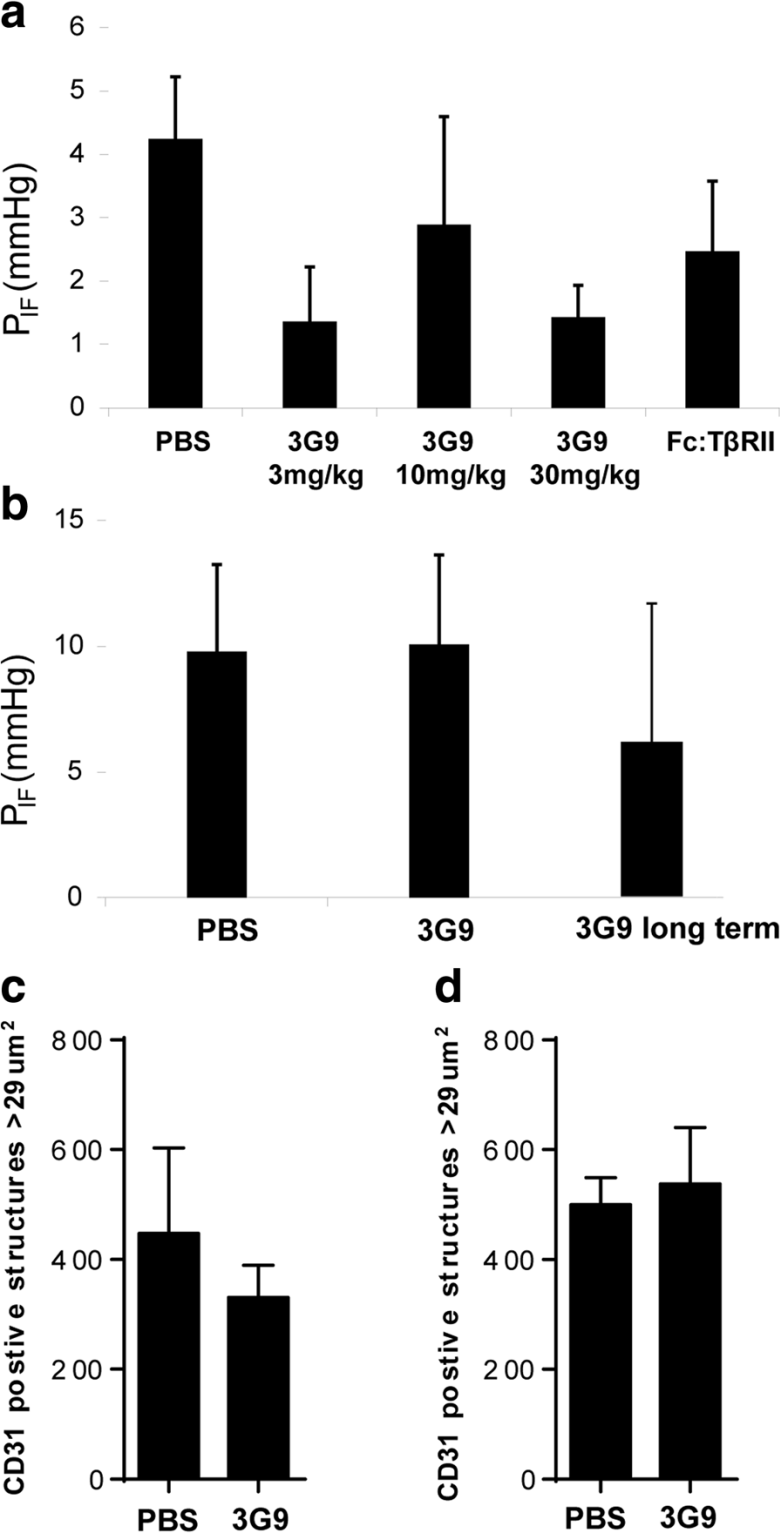
**a** Interstitial fluid pressure (P_IF_) in KAT-4 carcinomas treated with 3G9 at 3 (*n* = 8), 10 (*n* = 9), or 30 (*n* = 9) mg/kg, or with Fc:TβRII (10 mg/kg) (*n* = 9). PBS was used as negative control (*n* = 7). **b** Interstitial fluid pressure (P_IF_) in Capan-2 tumors treated three times with 3G9 (*n* = 5) or with 3G9 long term (*n* = 6) at10 mg/kg. PBS was used as control (*n* = 11). **c** CD31-stained structures with an area > 29 μm^2^ to avoid potential non-vascular CD31 positive structures in PBS- (*n* = 7) and 3G9-treated (*n* = 3) KAT-4 carcinomas. **d** CD31-stained structures measured as in C, but in PBS- (*n* = 4) and 3G9-treated (*n* = 5) Capan-2 carcinomas. Error bars are standard deviations

We next investigated the role of α_V_β_6_-mediated activation of latent TGF-β in maintaining a high P_IF_ in experimental carcinoma. KAT-4 carcinoma-bearing mice were treated with 3G9 at doses of 3, 10 and 30 mg/kg of body weight for 10 days. P_IF_, measured at day 11, was significantly lower in all treatment groups (*p* < 0.008 for all three groups, *n* = 10) (Fig. 5a). Administration of 10 mg/kg of Fc:TβRII at days 1 and 7 also reduced P_IF_ in accordance with previous reports [37] (*p* < 0.005) (Fig. 5a). In Capan-2 carcinomas P_IF_ values were generally higher than in KAT-4 carcinomas and had a much larger inter-tumor variation. Treatment of Capan-2 carcinomas with 3G9 administered at a dose of 10 mg/kg had no significant effect on P_IF_ (Fig. 5b). Treatment of Capan-2 carcinomas with 3G9 according to the extended protocol with injections at days 1, 3, 7, 10 and 14 resulted, however, in a trend for a lowered P_IF_ (measured on day 19, Fig. 5b), although this effect did not reach significance due to the large inter-tumoral variation (Fig. 5b). No significant correlation between tumor size and P_IF_ was observed in any of the two carcinoma models (Additional file 4). Staining with the endothelial cell marker CD31 did not show any significant effect of 3G9 on blood vessel density in either Capan-2 or KAT-4 carcinomas (Fig. 5c, d).

## Discussion

We hypothesized that α_V_β_6_ integrin plays an essential role in the activation of latent TGF-β in carcinoma and thereby participates in processes dependent on TGF-β signaling. To test this hypothesis we utilized the α_V_β_6_ integrin-blocking antibody 3G9 in two experimental models of carcinoma - KAT-4 (HT-29) colorectal adenocarcinoma and Capan-2 ductal pancreatic carcinoma. Our data indicate that α_V_β_6_ integrin is involved in latent TGF-β activation, but also that the dependency on this pathway may differ between different carcinoma types.

The pivotal role of α_V_β_6_ integrin in latent TGF-β activation in carcinoma is concluded from several lines of evidence: firstly, KAT-4 cells activated latent TGF-β and transferred the active growth factor to target cells by an α_V_β_6_-dependent process; Capan-2 cells, however, were unable to activate latent TGF-β unless they were co-cultured with human F99 fibroblasts. The latter effect was dependent on α_V_β_6_ integrin since it was blocked by 3G9 IgG and not by peptides that inhibit the function of α_V_β_5_ and α_V_β_3_ integrins. Secondly, the blocking of either α_V_β_6_ integrin or TGF-β1 and -β3 resulted in thinner collagen fibril structure. This phenotypic response was evident in KAT-4 carcinomas after a 10-day treatment period with 3G9 IgG (akin to the effects of the specific TGF-β1 and -β3 inhibitor Fc:TβRII [18, 37]), the same effect was observed in the Capan-2 model albeit after a longer treatment period with 3G9 IgG.

Whereas blockage of TGF-β1 and -β3 in the Capan-2 carcinoma model resulted in thinner collagen fibrils after a 10-day treatment period a prolonged 19-day treatment period was needed to achieve the same effect by 3G9 IgG. The reason for this discrepancy is not clear. 3G9 IgG and Fc:TβRII have similar molecular weights and structures and it is unlikely that the difference in sensitivity to 3G9 IgG and Fc:TβRII can be attributed to disparities in the uptake and distribution in the carcinoma tissue. In our microarray study we observed that Capan-2 cells overexpressed *LOXL4* and *TGM2,* both functioning in cross-linking of extracellular matrix proteins [38, 39], which could affect the mechanical forces generated by integrin pulling on the matrix-embedded latent TGF-β complex. Capan-2, or indeed even other desmoplastic carcinomas, may therefore propagate a distinct ECM structure that enhances the α_V_β_6_-dependent latent TGF-β activation pathway [40] thereby requiring a prolonged treatment period with repeated injection of 3G9 IgG. We also observed an increased abundance of several ECM-related transcripts in Capan-2 cells, transcripts that could contribute to the extent of α_V_β_6_-dependent TGF-β activation. A higher expression of *PLAT* and *PLAU* genes, encoding tissue plasminogen activator and urokinase-type plasminogen activator, could lead to a more pronounced conversion of plasminogen to plasmin, a process that has been implicated in enhanced latent TGF-β activation [9].

Activation of latent TGF-β by both KAT-4 and Capan-2/F99 were dependent on the downstream effector of Rho GTPase, ROCK. This is in agreement with published reports [36] and also with the notion that generation of tension is important for integrin α_V_β_6_-directed activation of latent TGF-β [9, 40]. It is thus possible that the different abilities of KAT-4 and Capan-2 cells to activate latent TGF-β stem from differences in capacities to generate cellular tension. For example, we observed that the latent TGF-β was activated when Capan-2 cells were co-cultured with F99 fibroblasts, which could be explained by the F99 fibroblasts supplying tensile forces to adjacent Capan-2 cells.

With regards to cultured KAT-4 cells’ ability to autonomously activate latent TGF-β using the α_V_β_6_ integrin we observed that the single-cell KAT-4 cultures express higher levels of *TSPAN8* - a member of tetraspanin proteins that co-localize with integrins and influence cellular motility [41]. It is conceivable that this protein may interact with α_V_β_6_ integrin and thus influence the activation of latent TGF-β.

Previous studies of experimental carcinomas have revealed a correlation between P_IF_ and specific collagen network properties like collagen fibril density, fibril structure or network architecture [18, 42, 43]. Treatment of experimental carcinoma with imatinib (STI571, Glivec) significantly lowers P_IF_, increases the extracellular fluid volume and enhances the dynamic exchange of solutes between tumor interstitium and blood [44, 45]. These effects are paralleled by a decrease in average collagen fibril diameter and an increase in collagen turnover in stroma ECM [45]. The effects of imatinib resemble those of inhibitors of the α_V_β_6_/TGF-β signaling axis. Imatinib selectively inhibits the tyrosine kinase c-Abl [46] and c-Abl is stimulated by TGF-β in a non-Smad dependent pathway [47]. It is possible that inhibition of c-Abl constitutes a convergence point for the effects on carcinoma stroma exerted by inhibitors of the α_V_β_6_/TGF-β signaling axis and imatinib. We have observed that imatinib abolished TGF β-elicited activation of the PAI-1 promotor in the mink-lung epithelial cells (Additional file 5). These data show that imatinib can inhibit at least some aspects of TGF-β-elicited signaling, aspects that may be of importance for the build-up and maintenance of pathologic stroma in carcinoma.

In the ECM, collagen together with hyaluronan and other glycosaminoglycans (GAG) form a functional and integrated matrix that determines fluid transport properties through tissues [48]. Interference either by alterations or depletions of the collagen or GAG components will change the physiological properties, including P_IF_. The data reported herein, emphasizing the importance of the collagen network, are clearly in accordance with this model, as collagen provides the framework for GAGs. In the less collagenous KAT-4 model P_IF_ was reduced as collagen fibril diameter decreased. Capan-2 tumors had a trend towards decreased P_IF_; these tumors, however, are more collagenous, which may reduce the hydration of GAGs in spite of a reduced fibril diameter, even after an extended treatment period.

## Conclusion

TGF-βs have dual roles during tumor development. In early malignant lesions they inhibit cell proliferation while during later stages they promote carcinoma progression by stimulating epithelial-to-mesenchymal transition, suppressing immune surveillance and aiding in the formation of a permissive microenvironment [6]. Notwithstanding the complexities of TGF-β pathway modulation our study shows that in selected carcinomas an inhibition of α_V_β_6_/TGF-β signaling axis alters the tumor stroma towards an alteration of the collagen network structure that previously has been shown to be associated with improved drug efficacy; the present study could open new venues in the research to improve chemotherapeutic efficacy.

## Additional files

Additional file 1: FACS analysis showing that both KAT-4 and Capan-2 tumor cells expressed α_v_β_6_ integrin on their cell surfaces (black peaks). IgG is negative control (grey peaks). (TIF 144 kb)

Additional file 2: TGF-β activation and inhibition in fibroblast and Capan-2 co-cultured with MLEC. Data was analyzed with Student’s t-test. Error bars are standard deviations. **A)** TGF-β activation inhibition with Cilengitide (0 to 10 μM). **B)** TGF-β activation inhibition with cRGD peptide (0 to 10 μM). **C)** Effects on TGF-β activation by co-culturing KAT-4 cells (Kat4, K) or Capan-2 (Capan2, C) together with MLEC reporter cells (M) on plastic dishes coated with human plasma fibronectin (HFN) or human plasma vitronectin (Vn). (TIF 8227 kb)

Additional file 3: Differential gene expression in KAT-4 vs. Capan-2 single-cell cultures. Transcripts with log_2_ fold change > 4 are shown and annotated using GO terms or experimental evidence reported in Uniprot. Positive fold change means higher transcript levels in Capan-2, negative fold change means higher transcript levels in KAT-4. (PDF 44 kb)

Additional file 4: Relationships between carcinoma size (weight) and interstitial fluid pressure (P_IF_). P_IF_ values were recorded in PBS-treated (control) KAT-4 (*n* = 9) and Capan-2 (*n* = 14) carcinomas, the carcinomas were excised and finally weighed. The data are partly collected from carcinomas showed in in Fig. 5. (PDF 40 kb)

Additional file 5: Effects of the tyrosine kinase inhibitor imatinib (STI571) on TGF β-induced PAI-1 expression in mink lung epithelial cells (MLEC) was recorded as detailed in Material and Methods. The signal recorded after stimulation with 10 nM TGF-β was normalized to 1 (*n* = 6). The effect on TGF-β-elicited signaling by 10 μM STI571 is shown (*p* < 0.0001, *n* = 3). Error bars +/− SD. All reagents were added simultaneously to the MLEC cultures at the start of the incubations. (TIF 393 kb)

## Abbreviations

CM: Conditioned media
ECM: Extracellular matrix
Fc:TβRII: soluble TGF-β receptor type II IgG_2A_ fusion protein
GAG: Glycosaminoglycans
MLEC: Mink Lung Epithelial cells
PAI-1: Plasminogen-activator inhibitor-1
P_IF_: Interstitial fluid pressure
ROCK: Rho-associated coiled coil forming protein serine/threonine kinase
TEM: Transmission electron microscopy
TGF-β: Transforming growth factor-β; tissue plasminogen activator
uPA: urokinase-type plasminogen activator

## References

[1] Nagy JA, Brown LF, Senger DR, Lanir N, Van de Water L, Dvorak AM, Dvorak HF (1989). Pathogenesis of tumor stroma generation: a critical role for leaky blood vessels and fibrin deposition. Biochim Biophys Acta.

[2] Hanahan D, Weinberg RA (2011). Hallmarks of cancer: the next generation. Cell.

[3] Kalluri R (2016). The biology and function of fibroblasts in cancer. Nat Rev Cancer.

[4] Heldin CH, Rubin K, Pietras K, Ostman A (2004). High interstitial fluid pressure - an obstacle in cancer therapy. Nat Rev Cancer.

[5] Drake LE, Macleod KF (2014). Tumour suppressor gene function in carcinoma-associated fibroblasts: from tumour cells via EMT and back again?. J Pathol.

[6] Pickup M, Novitskiy S, Moses HL (2013). The roles of TGFbeta in the tumour microenvironment. Nat Rev Cancer.

[7] Roberts AB, Sporn MB, Assoian RK, Smith JM, Roche NS, Wakefield LM, Heine UI, Liotta LA, Falanga V, Kehrl JH (1986). Transforming growth factor type beta: rapid induction of fibrosis and angiogenesis in vivo and stimulation of collagen formation in vitro. Proc Natl Acad Sci U S A.

[8] Blobe GC, Schiemann WP, Lodish HF (2000). Role of transforming growth factor beta in human disease. N Engl J Med.

[9] Robertson IB, Rifkin DB. Regulation of the bioavailability of TGF-beta and TGF-beta-related proteins. Cold Spring Harb Perspect Biol. 2016;8(6). 10.1101/cshperspect.a021907. 10.1101/cshperspect.a021907 PMC4888822 27252363

[10] Aluwihare P, Mu Z, Zhao Z, Yu D, Weinreb PH, Horan GS, Violette SM, Munger JS (2009). Mice that lack activity of alphavbeta6- and alphavbeta8-integrins reproduce the abnormalities of Tgfb1- and Tgfb3-null mice. J Cell Sci.

[11] Sheppard D (2005). Integrin-mediated activation of latent transforming growth factor beta. Cancer Metastasis Rev.

[12] Van Aarsen LA, Leone DR, Ho S, Dolinski BM, McCoon PE, LePage DJ, Kelly R, Heaney G, Rayhorn P, Reid C (2008). Antibody-mediated blockade of integrin alpha v beta 6 inhibits tumor progression in vivo by a transforming growth factor-beta-regulated mechanism. Cancer Res.

[13] Khan Z, Marshall JF (2016). The role of integrins in TGFbeta activation in the tumour stroma. Cell Tissue Res.

[14] Elayadi AN, Samli KN, Prudkin L, Liu YH, Bian A, Xie XJ, Wistuba II, Roth JA, McGuire MJ, Brown KC (2007). A peptide selected by biopanning identifies the integrin alphavbeta6 as a prognostic biomarker for nonsmall cell lung cancer. Cancer Res.

[15] Tod J, Hanley CJ, Morgan MR, Rucka M, Mellows T, Lopez MA, Kiely P, Moutasim KA, Frampton SJ, Sabnis D (2017). Pro-migratory and TGF-beta-activating functions of alphavbeta6 integrin in pancreatic cancer are differentially regulated via an Eps8-dependent GTPase switch. J Pathol.

[16] Dutta A, Li J, Fedele C, Sayeed A, Singh A, Violette SM, Manes TD, Languino LR (2015). alphavbeta6 integrin is required for TGFbeta1-mediated matrix metalloproteinase2 expression. Biochem J.

[17] Dahlman T, Lammerts E, Bergstrom D, Franzen A, Westermark K, Heldin NE, Rubin K (2002). Collagen type I expression in experimental anaplastic thyroid carcinoma: regulation and relevance for tumorigenicity. Int J Cancer.

[18] Oldberg A, Kalamajski S, Salnikov AV, Stuhr L, Morgelin M, Reed RK, Heldin NE, Rubin K (2007). Collagen-binding proteoglycan fibromodulin can determine stroma matrix structure and fluid balance in experimental carcinoma. Proc Natl Acad Sci U S A.

[19] Salnikov AV, Roswall P, Sundberg C, Gardner H, Heldin NE, Rubin K (2005). Inhibition of TGF-beta modulates macrophages and vessel maturation in parallel to a lowering of interstitial fluid pressure in experimental carcinoma. Lab Investig.

[20] Lohr M, Schmidt C, Ringel J, Kluth M, Muller P, Nizze H, Jesnowski R (2001). Transforming growth factor-beta1 induces desmoplasia in an experimental model of human pancreatic carcinoma. Cancer Res.

[21] Weinreb PH, Simon KJ, Rayhorn P, Yang WJ, Leone DR, Dolinski BM, Pearse BR, Yokota Y, Kawakatsu H, Atakilit A (2004). Function-blocking integrin alphavbeta6 monoclonal antibodies: distinct ligand-mimetic and nonligand-mimetic classes. J Biol Chem.

[22] Pfaff M, Tangemann K, Muller B, Gurrath M, Muller G, Kessler H, Timpl R, Engel J (1994). Selective recognition of cyclic RGD peptides of NMR defined conformation by alpha IIb beta 3, alpha V beta 3, and alpha 5 beta 1 integrins. J Biol Chem.

[23] Goodman SL, Holzemann G, Sulyok GA, Kessler H (2002). Nanomolar small molecule inhibitors for alphav(beta)6, alphav(beta)5, and alphav(beta)3 integrins. J Med Chem.

[24] Miekka SI, Ingham KC, Menache D (1982). Rapid methods for isolation of human plasma fibronectin. Thromb Res.

[25] Yatohgo T, Izumi M, Kashiwagi H, Hayashi M (1988). Novel purification of vitronectin from human plasma by heparin affinity chromatography. Cell Struct Funct.

[26] Abe M, Harpel JG, Metz CN, Nunes I, Loskutoff DJ, Rifkin DB (1994). An assay for transforming growth factor-beta using cells transfected with a plasminogen activator inhibitor-1 promoter-luciferase construct. Anal Biochem.

[27] Ain KB, Taylor KD (1994). Somatostatin analogs affect proliferation of human thyroid carcinoma cell lines *in vitro*. J Clin Endocrinol Metab.

[28] Schweppe RE, Klopper JP, Korch C, Pugazhenthi U, Benezra M, Knauf JA, Fagin JA, Marlow LA, Copland JA, Smallridge RC (2008). Deoxyribonucleic acid profiling analysis of 40 human thyroid cancer cell lines reveals cross-contamination resulting in cell line redundancy and misidentification. J Clin Endocrinol Metab.

[29] Hakelius M, Koskela A, Ivarsson M, Grenman R, Rubin K, Gerdin B, Nowinski D (2013). Keratinocytes and head and neck squamous cell carcinoma cells regulate urokinase-type plasminogen activator and plasminogen activator inhibitor-1 in fibroblasts. Anticancer Res.

[30] Rubin K, Sjoquist M, Gustafsson AM, Isaksson B, Salvessen G, Reed RK (2000). Lowering of tumoral interstitial fluid pressure by prostaglandin E(1) is paralleled by an increased uptake of (51)Cr-EDTA. Int J Cancer.

[31] Berg RA (1982). Determination of 3- and 4-hydroxyproline. Methods Enzymol.

[32] Ohtani O, Ushiki T, Taguchi T, Kikuta A (1988). Collagen fibrillar networks as skeletal frameworks: a demonstration by cell-maceration/scanning electron microscope method. Arch Histol Cytol.

[33] Saeed AI, Sharov V, White J, Li J, Liang W, Bhagabati N, Braisted J, Klapa M, Currier T, Thiagarajan M (2003). TM4: a free, open-source system for microarray data management and analysis. Biotechniques.

[34] Love MI, Huber W, Anders S (2014). Moderated estimation of fold change and dispersion for RNA-seq data with DESeq2. Genome Biol.

[35] Fontana L, Chen Y, Prijatelj P, Sakai T, Fassler R, Sakai LY, Rifkin DB (2005). Fibronectin is required for integrin alphavbeta6-mediated activation of latent TGF-beta complexes containing LTBP-1. FASEB J.

[36] Giacomini MM, Travis MA, Kudo M, Sheppard D (2012). Epithelial cells utilize cortical actin/myosin to activate latent TGF-beta through integrin alpha(v)beta(6)-dependent physical force. Exp Cell Res.

[37] Lammerts E, Roswall P, Sundberg C, Gotwals PJ, Koteliansky VE, Reed RK, Heldin NE, Rubin K (2002). Interference with TGF-beta1 and -beta3 in tumor stroma lowers tumor interstitial fluid pressure independently of growth in experimental carcinoma. Int J Cancer.

[38] Choi SK, Kim HS, Jin T, Moon WK (2017). LOXL4 knockdown enhances tumor growth and lung metastasis through collagen-dependent extracellular matrix changes in triple-negative breast cancer. Oncotarget.

[39] Collighan RJ, Griffin M (2009). Transglutaminase 2 cross-linking of matrix proteins: biological significance and medical applications. Amino Acids.

[40] Wipff PJ, Hinz B (2008). Integrins and the activation of latent transforming growth factor beta1 - an intimate relationship. Eur J Cell Biol.

[41] Zoller M (2009). Tetraspanins: push and pull in suppressing and promoting metastasis. Nat Rev Cancer.

[42] Gade TP, Buchanan IM, Motley MW, Mazaheri Y, Spees WM, Koutcher JA (2009). Imaging intratumoral convection: pressure-dependent enhancement in chemotherapeutic delivery to solid tumors. Clin Cancer Res.

[43] Torosean S, Flynn B, Axelsson J, Gunn J, Samkoe KS, Hasan T, Doyley MM, Pogue BW (2013). Nanoparticle uptake in tumors is mediated by the interplay of vascular and collagen density with interstitial pressure. Nanomedicine.

[44] Klosowska-Wardega A, Hasumi Y, Burmakin M, Ahgren A, Stuhr L, Moen I, Reed RK, Rubin K, Hellberg C, Heldin CH (2009). Combined anti-angiogenic therapy targeting PDGF and VEGF receptors lowers the interstitial fluid pressure in a murine experimental carcinoma. PLoS One.

[45] Olsson PO, Gustafsson R, In ‘t Zandt R, Friman T, Maccarana M, Tykesson E, Oldberg A, Rubin K, Kalamajski S (2016). The tyrosine kinase inhibitor Imatinib augments extracellular fluid exchange and reduces average collagen fibril diameter in experimental carcinoma. Mol Cancer Ther.

[46] Buchdunger E, Cioffi CL, Law N, Stover D, Ohno-Jones S, Druker BJ, Lydon NB (2000). Abl protein-tyrosine kinase inhibitor STI571 inhibits in vitro signal transduction mediated by c-kit and platelet-derived growth factor receptors. J Pharmacol Exp Ther.

[47] Bhattacharyya S, Ishida W, Wu M, Wilkes M, Mori Y, Hinchcliff M, Leof E, Varga J (2009). A non-Smad mechanism of fibroblast activation by transforming growth factor-beta via c-Abl and Egr-1: selective modulation by imatinib mesylate. Oncogene.

[48] Levick JR (1987). Flow through interstitium and other fibrous matrices. Q J Exp Physiol.

[49] Edgar R, Domrachev M, Lash AE (2002). Gene expression omnibus: NCBI gene expression and hybridization array data repository. Nucleic Acids Res.

