# Supplementary figures and images for: Inhibition of integrin αVβ6 changes fibril thickness of stromal collagen in experimental carcinomas
Source: Cell Commun Signal. 2018 Jul 2;16:36. doi: 10.1186/s12964-018-0249-7 (PMC6027735; doi:10.1186/s12964-018-0249-7)

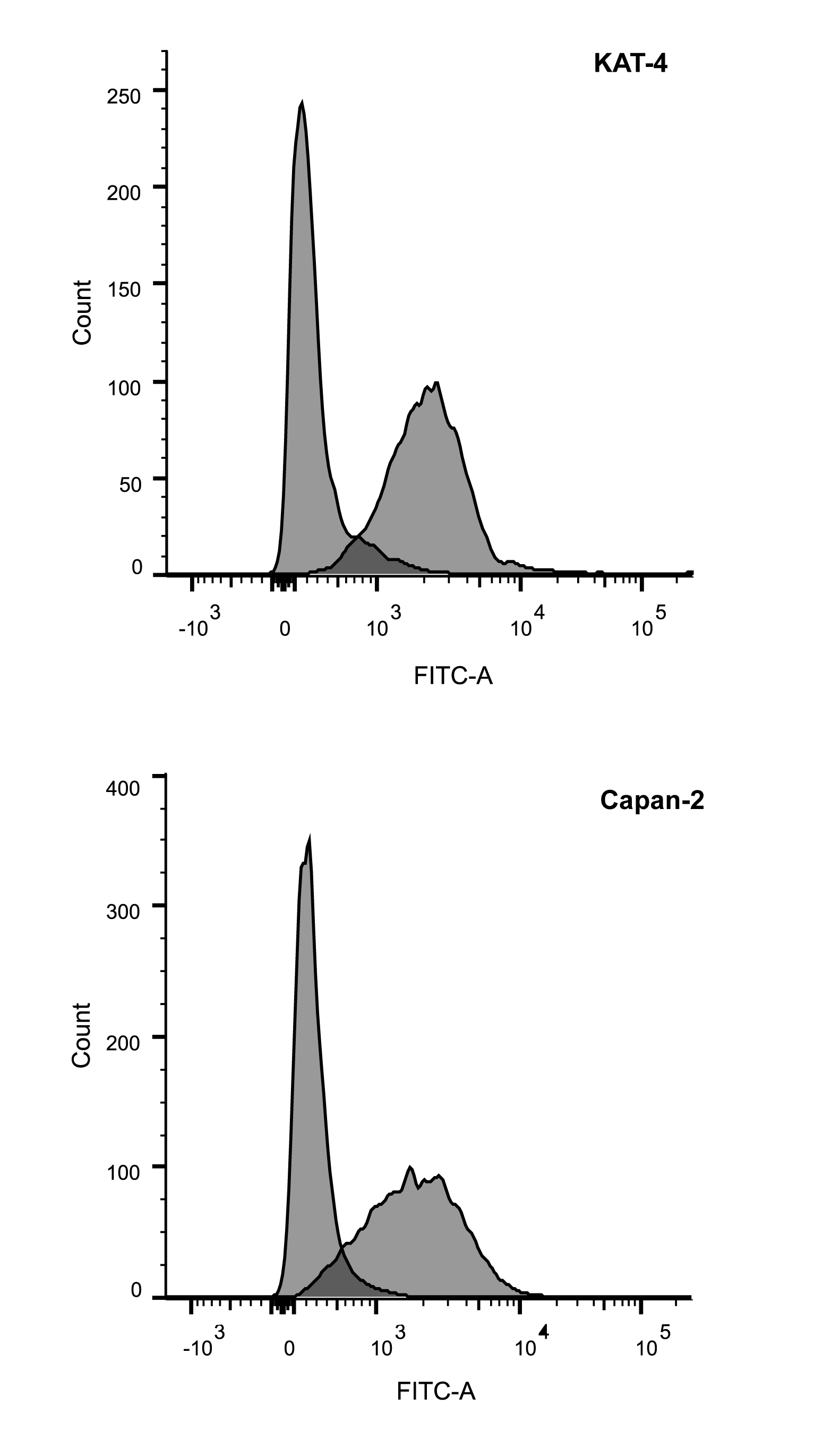

Supplement: Supplementary file 1 — FACS analysis showing that both KAT-4 and Capan-2 tumor cells expressed αvβ6 integrin on their cell surfaces (black peaks). IgG is negative control (grey peaks). (TIF 144 kb) [file 12964_2018_249_MOESM1_ESM.tif]

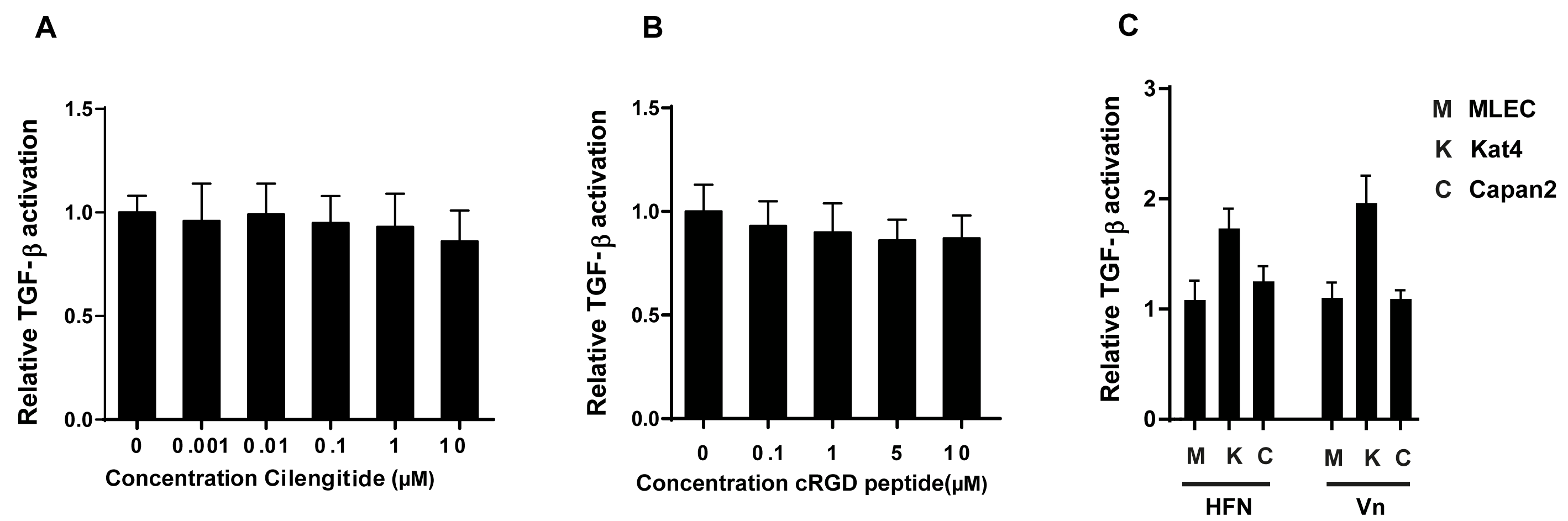

Supplement: Supplementary file 2 — TGF-β activation and inhibition in fibroblast and Capan-2 co-cultured with MLEC. Data was analyzed with Student’s t-test. Error bars are standard deviations. A) TGF-β activation inhibition with Cilengitide (0 to 10 μM). B) TGF-β activation inhibition with cRGD peptide (0 to 10 μM). C) Effects on TGF-β activation by co-culturing KAT-4 cells (Kat4, K) or Capan-2 (Capan2, C) together with MLEC reporter cells (M) on plastic dishes coated with human plasma fibronectin (HFN) or human plasma vitronectin (Vn). (TIF 8227 kb) [file 12964_2018_249_MOESM2_ESM.tif]

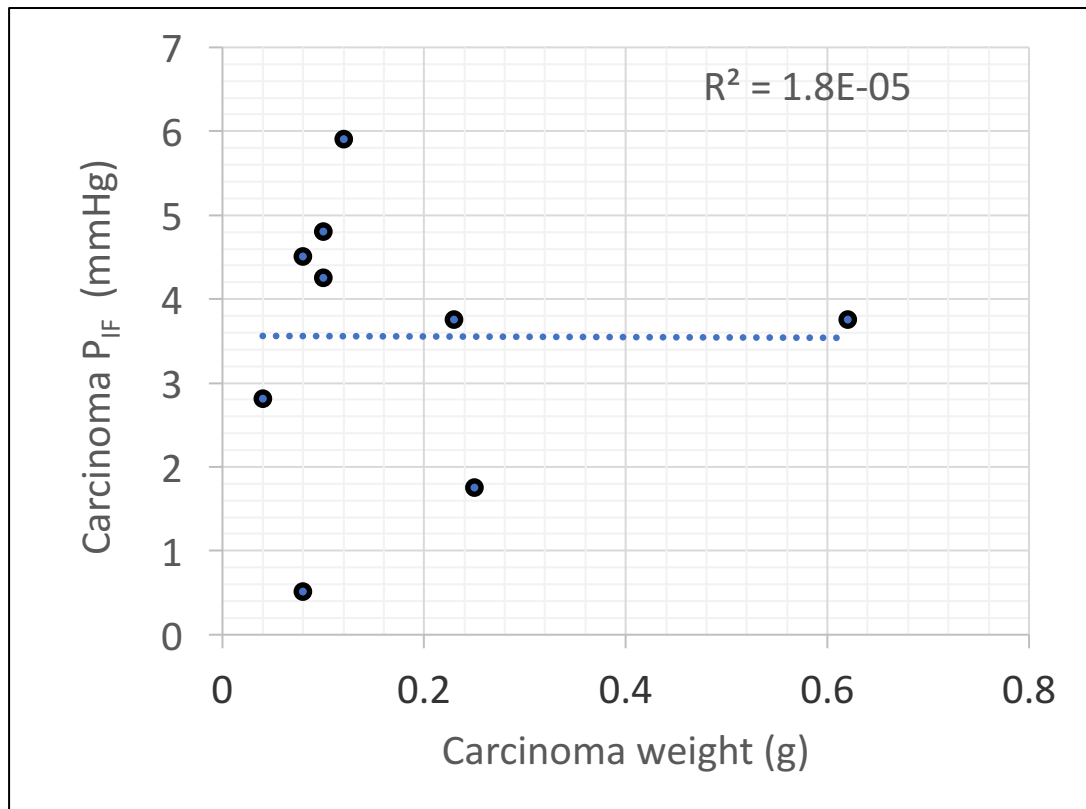

KAT-4

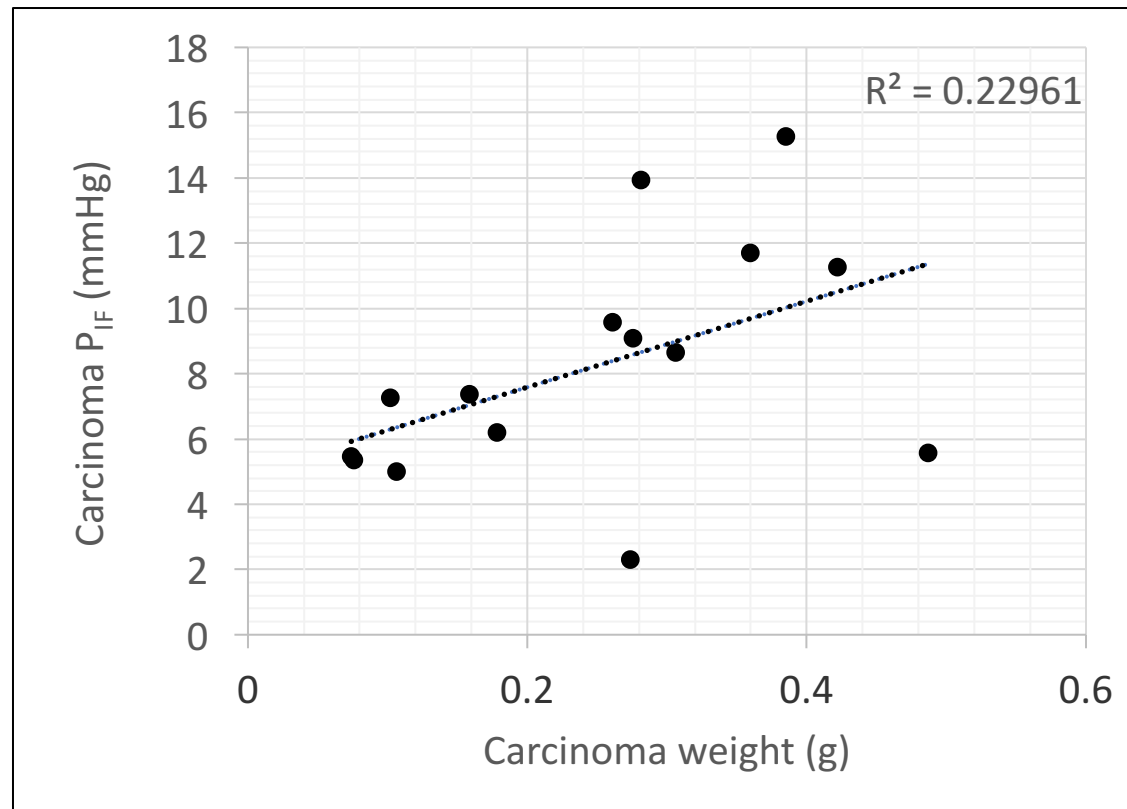

Capan-2

Supplement: Supplementary file 4 — Relationships between carcinoma size (weight) and interstitial fluid pressure (PIF). PIF values were recorded in PBS-treated (control) KAT-4 (n = 9) and Capan-2 (n = 14) carcinomas, the carcinomas were excised and finally weighed. The data are partly collected from carcinomas showed in in Fig. 5. (PDF 40 kb) [file 12964_2018_249_MOESM4_ESM.pdf]

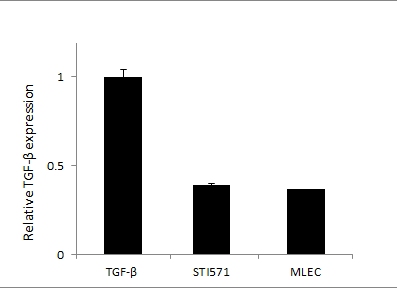

Supplement: Supplementary file 5 — Effects of the tyrosine kinase inhibitor imatinib (STI571) on TGF β-induced PAI-1 expression in mink lung epithelial cells (MLEC) was recorded as detailed in Material and Methods. The signal recorded after stimulation with 10 nM TGF-β was normalized to 1 (n = 6). The effect on TGF-β-elicited signaling by 10 μM STI571 is shown (p < 0.0001, n = 3). Error bars +/− SD. All reagents were added simultaneously to the MLEC cultures at the start of the incubations. (TIF 393 kb) [file 12964_2018_249_MOESM5_ESM.tif]
