# Supplementary material for: Inhibition of integrin αVβ6 changes fibril thickness of stromal collagen in experimental carcinomas
Source: Cell Commun Signal. 2018 Jul 2;16:36. doi: 10.1186/s12964-018-0249-7 (PMC6027735; doi:10.1186/s12964-018-0249-7)
Supplement: Supplementary file 3 — Differential gene expression in KAT-4 vs. Capan-2 single-cell cultures. Transcripts with log2 fold change > 4 are shown and annotated using GO terms or experimental evidence reported in Uniprot. Positive fold change means higher transcript levels in Capan-2, negative fold change means higher transcript levels in KAT-4. (PDF 44 kb) [file 12964_2018_249_MOESM3_ESM.pdf]

KAT-4 vs. Capan-2 cell differential gene expression. The table shows transcripts that differ by log_2-fold change > 4, where positive values indicate higher quantity in Capan-2 and negative values higher quantity in KAT-4 cells. Transcripts were annotated using GO terms or experimental evidence presented in Uniprot.

**Annotated function**	**Transcript**	**log_2 fold change**
Cellular adhesion	*TACSTD2*	7.0
	MSLN	5.1
	MUC1	4.2
	BSG	-4.9
	TSPAN8	-6.2
Cell proliferation or apoptosis, cell signaling	*RARRES1*	5.3
	RARRES3	4.3
	IGFBP3	4.2
	PLAC8	4.6
	NGFRAP1	4.5
	EMP1	4.1
Morphogenesis	*CST6*	4.3
Serine protease/inhibitors	*KLK6*	6.4
	SPINK1	-5.6
Protein cross-linking	*TGM2*	5.7
	LOXL4	4.5
Plasminogen-to-plasmin conversion	*PLAT*	5.2
	PLAU	4.5
Ubiquitination	*UBE2L6*	4.9
Cytoskeleton	*KRT7*	4.2
Immune response-related	*HLA-B*	5.0
	IFITM3	4.5
	CD24	-6.4
	CD74	4.5
	LCN2	4.3
	LYZ	-4.0
Metabolism	*AKR1B1*	5.4
	AKR1B10	-6.0
	ALDH1A3	5.0
	HSD17B2	-4.2
	HMGCS2	-4.4
	LDHB	-5.3
Digestion	*TFF2*	4.8
	TFF3	-4.0
